# Bioprocess scale‐up/down as integrative enabling technology: from fluid mechanics to systems biology and beyond

**DOI:** 10.1111/1751-7915.12803

**Published:** 2017-08-14

**Authors:** Frank Delvigne, Ralf Takors, Rob Mudde, Walter van Gulik, Henk Noorman

**Affiliations:** ^1^ TERRA Research Center Microbial Processes and Interactions (MiPI) University of Liège Liège Belgium; ^2^ Institute of Biochemical Engineering University of Stuttgart Stuttgart Germany; ^3^ Transport Phenomena Section Department of Chemical Engineering Delft University of Technology Delft The Netherlands; ^4^ Department of Biotechnology Delft University of Technology Delft The Netherlands; ^5^ DSM Biotechnology Center Delft The Netherlands

## Abstract

Efficient optimization of microbial processes is a critical issue for achieving a number of sustainable development goals, considering the impact of microbial biotechnology in agrofood, environment, biopharmaceutical and chemical industries. Many of these applications require scale‐up after proof of concept. However, the behaviour of microbial systems remains unpredictable (at least partially) when shifting from laboratory‐scale to industrial conditions. The need for robust microbial systems is thus highly needed in this context, as well as a better understanding of the interactions between fluid mechanics and cell physiology. For that purpose, a full scale‐up/down computational framework is already available. This framework links computational fluid dynamics (CFD), metabolic flux analysis and agent‐based modelling (ABM) for a better understanding of the cell lifelines in a heterogeneous environment. Ultimately, this framework can be used for the design of scale‐down simulators and/or metabolically engineered cells able to cope with environmental fluctuations typically found in large‐scale bioreactors. However, this framework still needs some refinements, such as a better integration of gas–liquid flows in CFD, and taking into account intrinsic biological noise in ABM.

## Bioprocess scale‐up/down: historical perspectives and computational framework

Scale‐up of fermentation processes in the biotech industry is a critical step in bringing bioprocess and bioproduct innovations to commercialization. This step is notorious for performance losses and delays in development. The scale‐down approach has been advocated as a smart way to minimize these issues. In principle, this approach consists of four interconnected activities:

- Detailed analysis of the large‐scale conditions, characterizing the dynamic environment witnessed by the microorganisms;
- As precise as possible translation of this environment to the laboratory‐scale, via design of scale‐down simulators;
- Tests at laboratory‐scale under conditions representative for large scale to find better combinations of strains and environmental conditions;
- Back‐translation of successful findings (e.g. more robust strains, less variable environments) to the large scale.

Already in the 1980s, benefits of the scale‐down approach have been demonstrated (Oosterhuis, 1983; Sweere and Mesters, 1988), although at that time, the level of precision was limited. Later on, early computational approaches (Euler–Euler modelling methods) have been added to capture large‐scale process details with higher resolution (Larsson *et al*., 1996; Enfors *et al*., 2001), also refining the scale‐down simulator design. One major step forward then was the introduction of computational methods that use the perspective from the microbial viewpoint, that is describing the lifelines of individual cells on their journey through the large‐scale bioreactor (Lapin *et al*., 2004). This is referred to as Euler–Lagrange or agent‐based modelling (ABM). In more recent years, the continuously advancing computer power and better insight into metabolic dynamics have enabled major resolution of the reactor content, in hundreds of thousands location points, with lifelines of millions individual cells (Haringa *et al*., 2017; Haringa *et al*., 2016). Today, this presents an opening to a different way of bioprocess engineering, not anymore based on gross simplification, but rather on brute force simulations of reality including much of its complexity.

Sound scale‐up/down strategies rely heavily on accurate description of the phenomena at the various scales. Hereby, it should be realized that the environment of the microorganisms changes drastically when scaling up or down (Oosterhuis, 1983). This is inherent to the fluid dynamics involved: the larger the scale, the more prominent the influence of turbulence. As fluid mechanics and in particular turbulence are nonlinear phenomena, a straightforward strategy cannot be formulated. Mixing of nutrient feeds, shear rates, mass transfer and transport of the microorganisms through the reactor are all influenced by the flow and turbulence. Computational fluid dynamics (CFD) addresses all these issues (Sweere and Mesters, 1988; Larsson *et al*., 1996). With this computational approach, we can accurately simulate the fluid flow in the reactor and track nutrient mixing. Moreover, we can simulate the journeys of a large number (10^5^–10^7^) of microorganisms through the bioreactor. In this way, we are able to study large‐scale fermentation processes through the eyes of the microorganisms (Enfors *et al*., 2001), taking into account the wide spread in scales in liquid flow, concentration gradients of nutrients and oxygen and can even assess the effect of cooling coils and other internals if needed. Furthermore, the effect of changes in the local surroundings of the microorganisms can be coupled to the performance of the cells, for example in terms of substrate uptake, growth and (by)‐product formation. To this end, compact reaction kinetic (CRK) models are used to keep track of the pools of the most important species inside the cell. In summary, this allows a coupling of the processes inside the cells in response to the ‘turbulent environment’ that they experience over time (Lapin *et al*., 2004).

**Figure 1:**
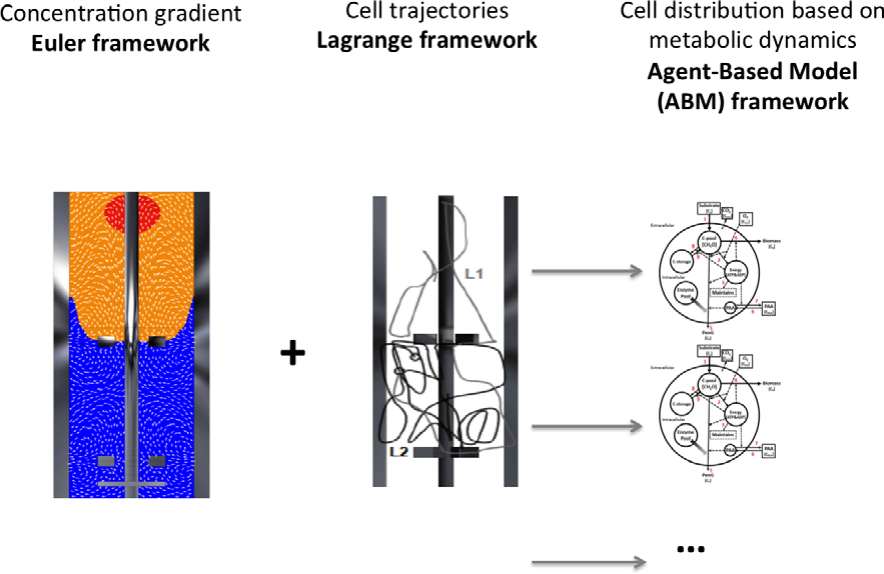
Computational framework used for scaling‐up/down studies. A concentration gradient flow field is simulated based on the numerical resolution of the Navier–Stokes equations based on a Eulerian reference. Cell trajectories can be simulated with the same model translated in a Lagrangian reference. Superposition leads to a cell lifelines distribution. An agent‐based model incorporating a relevant metabolic network is finally needed for the precise simulation of the environmental impact on the state of individual cells. The panel showing the Lagrangian reference has been adapted from Kuschel *et al*. (2017).

The basic idea of CFD is to solve the equations of motion of the fluid in the reactor (i.e. the Navier–Stokes equations). These equations describe conservation of mass and the balance of momentum in the fluid. In particular, the momentum balance is of a nonlinear nature, which prohibits analytical solutions for virtually all practical flows. This requires discretizing the mathematical equations and solving them numerically. However, there is a serious catch: when scaling up, the flow becomes more turbulent and a feature of turbulent flows is a wide span of scales both in space and in time. This span goes up so quickly when increasing the turbulence, that for industrial size equipment, even our biggest computers are not large enough to deal with it. Therefore, only the dominant scales in the flow are computed, while the small scales are modelled using a turbulence model (Haringa *et al*., 2017). Once this is established, the microorganisms can be tracked during their motion through the reactor (Figure 1). This is done in a Lagrangian way: a large collection of individual cells is tracked in the flowing liquid. Because the fluid is simulated as a continuum and the microorganisms as particles, this method is called Euler–Lagrange simulation. Enfors *et al*. (2001) pioneered this approach for industrial bioreactors. The organisms are so small that the drag forces exerted by the fluid completely govern their motion. Consequently, the microorganisms follow the motion of the fluid. But there is another catch here: only the large scale of the flow field is known. The effect of the smaller structures (i.e. all smaller scale eddies in the fluid) needs to be put in the simulation via a modelling approach. This is, for example, done via random walk approaches: the small‐scale structures act like a kind of diffusion on the motion of the microorganisms. This means that cells that would start at exactly the same location at exactly the same time would not follow the same trajectory. The consequence is that every time when a new position of a cell is computed (based on the computed fluid flow), a random side step is added that mimics the effect of the turbulence. By storing the positions of the cells as function of time, the ‘lifeline’ of each simulated cell is recorded (Enfors *et al*., 2001; Haringa *et al*., 2016; Kuschel *et al*., 2017).

The CFD approach outlined above results in tracking the positions of all simulated cells in the reactor as a function of time. At the same time, the local environment of each cell at each time is also computed. In the second step, the external world of the cell is coupled to the internal space in a dynamic, local way. The cell takes up nutrients and, possibly, oxygen depending on the local driving forces. Moreover, the cells influence the local driving forces via nutrient uptake. Finally, a cell has the ability to store metabolites and macromolecules (e.g. trehalose, glycogen) and thus survive periods of local starvation providing that it returns back to nutrient‐rich zones in sufficient time. This means that the internal metabolic processes should be simulated at the same time scale as the external mixing and transport. Currently, it is not possible to do that all from first principles. Luckily, that is also not needed. The fluid(s) flow field can be computed separately and serve as frozen input to the species and cell transport coupled to the internal metabolic processes. The reason for this is that the cells can be considered as tracers that do not modify the flow field (Oosterhuis *et al*., 1985).

## From dry‐laboratory to wet‐laboratory approach: designs and applications of scale‐down simulators

Despite the logic of the scale‐down approach, novel bioprocesses are still often developed and optimized first in small‐scale bioreactors (1–30 L) before they are transferred into large‐scale industrial conditions. In addition, rigorous modelling or computation approaches are scarce. Laboratory‐scale data represent the most important experimental proof for the performance of the process. Titres, productivities, product/substrate conversion efficiencies, product purities, etc., are not only used to design up‐ and downstream processing units but also to define the operating window of economic success in large‐scale production. The performance of laboratory‐scale fermentations usually defines the economic expectations of the industrial process. Any kind of large‐scale underperformance compared to the design criteria may push the process out of the economic operation window and may render it non‐viable. To increase the probability of success, tools to predict large‐scale performance either by simulating industrial conditions in so‐called scale‐down wet‐laboratory simulators or by dry‐laboratory simulation of large‐scale performance are highly welcome, but by and large these tools are not much applied, and certainly not in a quantitative way. The question is whether design of scale‐down simulators based on qualitative reasoning or without reference to large‐scale details makes sense for scale‐up research. At best perhaps, worst case options can be studied.

**Figure 2:**
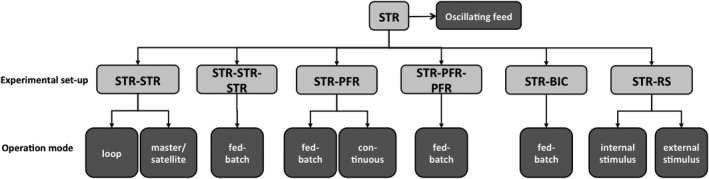
Scale‐down devices using the stirred tank reactor (STR) as an ideally mixed compartment connected to other STRs or plug‐flow reactors (PFR). Built‐in components (BIC) to reduce homogeneity also have been applied. Furthermore, large‐scale stimuli can be reflected in STR and monitored in rapid sampling (RS) approaches.

Stirred tank reactors (STR) represent the state of the art for wet‐laboratory tests. This may seem surprising considering that very large bioreactors are preferably designed as bubble columns or airlift reactors (Lübbert, 2000). However, laboratory‐scale STRs are regarded as ideally mixed subunits that can be linked together to mimic different metabolic regimes that are formed as a result of the circulation paths of cells entering and leaving different zones of large‐scale bioreactors (Figure 2).

First examples were published by Oosterhuis and Kossen (1983) and Oosterhuis *et al*. (1985) applying the STR‐STR set‐up for investigating the impact of different oxygen supplies in each STR. *Gluconobacter oxydans* was cycled between zones with high and low oxygen availability. Similar approaches were published by Lara *et al*. (2005), Lara *et al*. (2009) and recently by Kass *et al*. (2014) and Limberg *et al*. (2017) studying *Escherichia coli* and *Corynebacterium glutamicum* respectively. Alternatively, STR‐STR set‐ups could be operated in the so‐called master–slave mode, that is the ‘master’ reactor may be operated in fed‐batch mode sending control values of fermentation conditions to the ‘slave’ (or ‘satellite’) reactor. While running in parallel, the ‘satellite’ was labelled with ^13^C glucose thereby enabling detailed flux pattern analysis as a mirror of the ‘master’ performance (El Massaoudi *et al*., 2003). Similar set‐ups are also applied in industry to test impact of media components during piloting. Recently, a reactor cascade consisting of a main 100 L STR and two 1 L STRs arranged in a loop of the main reactor was published (Buchholz *et al*., 2014). Different partial pressures of dissolved CO_2_ were installed in the loop bioreactors to monitor fast transcriptional and metabolic responses to different CO_2_ pressures which occur in large‐scale production.

Probably, the most classical set‐up consists of an STR and a plug‐flow reactor installed in the loop of the STR. Gradients of substrates are imposed on cells cycling through the PFR with residence times that mirror large‐scale mixing conditions. First examples were published by George *et al*. (1993) and Neubauer *et al*. (1995) investigating the impact of oscillating glucose supply on *Saccharomyces cerevisiae* and *Escherichia coli* respectively. The set‐up is frequently applied by various groups typically simulating phenotypic performance of fed‐batch cultivations (Junne *et al*., 2011). Recently, the STR‐PFR approach was applied in continuous mode using the steady state without PFR as a reference for quantifying short‐ and long‐term transcriptional and metabolic responses of *E. coli* (Loffler *et al*., 2017; Simen *et al*., 2017). Furthermore, the concept of STR‐PFR has been expanded to STR‐PFR‐PFR by integrating a second PFR loop to fine tune the representation of multiple zones inside a large‐scale bioreactor (Lemoine *et al*., 2015). Whereas the aforementioned scale‐down devices needed external units to impose large‐scale stress conditions, Schilling *et al*. (1999) integrated perforated plates in an STR‐BIC (built‐in components) approach for deteriorating the mixing between different reactor zones. STR‐RS (rapid sampling) devices are used to monitor the short‐term metabolic dynamics after perturbations that mirror stimuli of large‐scale inhomogeneities. Observed metabolic dynamics serve for model identification finally to allow model‐predicted large‐scale performance (Tang *et al*., 2017). Stimuli may be imposed either internal or external of the STR. Cell sampling must be done rapidly accompanied by a fast inactivation of cellular metabolism. Examples of rapid sampling devices comprise heat exchangers (Theobald *et al*., 1993), frozen tubes (Weuster‐Botz, 1997), automated transportation belts (Schaefer *et al*., 1999), the BioScope technique (Visser *et al*., 2002) and many others.

In more recent scale‐down studies, omics‐based technologies have been applied with the aim to elucidate the physiological mechanisms responsible for loss of performance in large‐scale cultivation systems. In order to identify sets of ‘signature transcripts’ to monitor the nutritional status and possible stress conditions in large‐scale fermentations of *Saccharomyces cerevisiae,* transcriptome studies have been carried out under well‐defined conditions in aerobic and anaerobic chemostats under different nutrient limitations (C, N, P, S) (Boer *et al*., 2003; Tai *et al*., 2005). They identified a set of 155 specifically oxygen‐responsive genes and several sets specific for different macronutrient limitations. Transcriptome measurements have also been applied to investigate the physiological effects of dissolved oxygen gradients on recombinant *E. coli* in a scale‐down device (Lara *et al*., 2009). The measured transcriptional changes explained the observed alterations in stoichiometry and kinetics, as well as production of ethanol and organic acids. The observed differences in transcription levels between aerobic and anaerobic compartments of the scale‐down device indicated that *E. coli* responds rapidly to dissolved oxygen gradients.

Proteomic profiling of recombinant *E. coli* in high‐cell‐density fermentations revealed distinct physiological changes during the cultivation, for example synthesis of the phage shock protein A (PspA) appeared strongly correlated with product synthesis (Aldor *et al*., 2005). Controlled coexpression of PspA resulted in improvement of the product yield.

Metabolomics and protein measurements were applied to study the negative effect of substrate gradients on antibiotic production in *Penicillium chrysogenum* (de Jonge *et al*., 2011). To this end, feast–famine experiments were carried out in an intermittently fed chemostat. These conditions resulted in oscillations in intracellular adenosine nucleotide levels, possibly affecting the in vivo activity of l‐α‐(δ‐aminoadipyl)‐l‐α‐cysteinyl‐d‐α‐valine synthase (ACVS): the first enzyme of the penicillin biosynthesis pathway. In further feast–famine experiments, ^13^C‐based fluxomics and metabolomics revealed that substrate gradients resulted in extremely large oscillations in intermediates of central carbon metabolism and simultaneous synthesis and degradation of storage carbohydrates, resulting in additional ATP dissipation (de Jonge *et al*., 2014).

Exometabolome measurements were carried out to investigate the physiological effects of scale‐up of a high‐density fed‐batch process of *S. cerevisiae* (Fu *et al*., 2014) which revealed reduced oxygen availability at large scale resulting in overflow metabolism. Combined metabolome and proteome analysis indicated that hypoxia caused lower productivity upon scale‐up of a CHO bioprocess (Gao *et al*., 2016).

Proteome, transcriptome and metabolome analyses were carried out by Limberg *et al*. (2017) to identify the metabolic key mechanisms responsible for the tolerance of *Corynebacterium glutamicum* to oxygen and substrate gradients. The applied multiomics approach showed the metabolic flexibility of this organism to deal with inhomogeneities in large‐scale processes.

In these omics‐based approaches, the effects of scale‐up conditions on the physiology of the whole culture have been investigated; however, an additional aspect to be considered is possible heterogeneity of the culture itself, requiring single‐cell‐based techniques.

## A step forward: taking into account microbial individuality

Despite several efforts made at the experimental and computational level, bioprocess scale‐up/down is not only a tale of fluid mechanics and metabolite fluxes. Indeed, the microbial population itself is able to exhibit different diversification strategies. Phenotypic diversification naturally occurs in the environment (even under stable conditions) where this phenomenon typically leads to increased fitness of the whole microbial population (Martins and Locke, 2015), but also occurs in bioprocesses (Grunberger *et al*., 2014; Delvigne *et al*., 2014). Intracellular biochemical reactions are intrinsically noisy, considering the relatively low number of molecules involved (Hilfinger and Paulsson, 2011), leading to potential phenotypic heterogeneity in different microbial production hosts, for example through cell‐to‐cell variation in promoter expression, unequal transporter distribution and bet‐hedging following a diauxic shift (Binder *et al*., 2017). The fact that the microbial cell is intrinsically noisy has not yet been taken into account in the actual scale‐up/down computational framework (Lemoine *et al*., 2017). In practice, only a part of the extrinsic component of noise is actually taken into account by linking cell trajectories with effective metabolic fluxes distribution (Figure 1).

**Figure 3:**
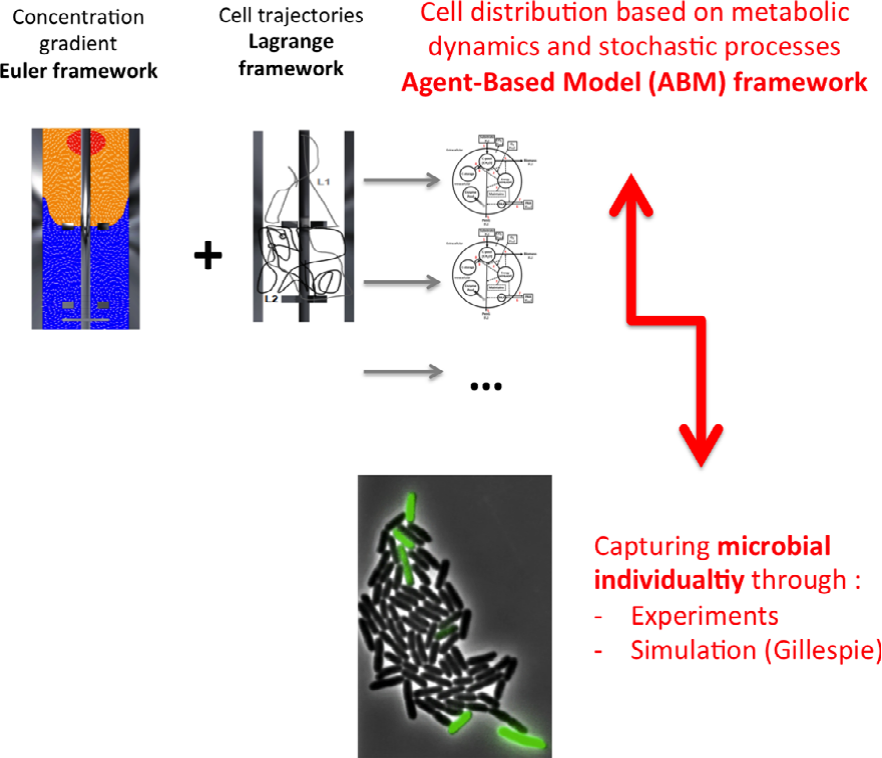
Experimental and modelling approaches are needed for incorporating microbial individuality in the scale‐up/down computational framework.

As for scale‐up/down studies, microbial population analysis relies both on computational and on experimental approaches. Experimental investigation of microbial phenotypic diversification in bioprocesses involves mainly the use of automated flow cytometry (Brognaux *et al*., 2013; Broger *et al*., 2011) or real‐time imaging in combination with tailored microfluidic cultivation devices (Grunberger *et al*., 2012; Dusny *et al*., 2012). Linking experimental single‐cell studies with *in silico* approaches allows for a better interpretation of microbial individuality in bioprocesses. As an example, automated flow cytometry has been used for validating a biological scaling law, linking protein expression to promoter noise in process‐related conditions (Baert *et al*., 2015). Stochastic simulation of intracellular biochemical reactions can be achieved through the use of the Gillespie algorithm (Gillespie, 1977), in a way that can be assimilated to an agent‐based model (ABM). Incorporating microbial individuality in the scale‐up/down computational framework could thus be achieved through the existing ABM (Figure 3). Indeed, stochastic processes can be directly incorporated in ABM (Hellweger *et al*., 2016). Such an approach has been used recently for simulating the dynamics of the *E. coli* tryptophan operon in a scale‐down reactor. The Gillespie algorithm representing trp‐dependent protein synthesis was directly incorporated in the ABM for this purpose (Niess *et al*., 2017).

## Scale‐up/down tools enable successful industrial implementation of microbial bioprocesses

The thorough application of the aforementioned tools provides a highly valuable (big) data basis mirroring the cellular responses to typical large‐scale stimuli. Data should be used to derive and validate proper models for *in silico* and *ab initio* prediction of large‐scale performances. Notably, these approaches are a useful tool to reduce manpower and money for tedious scale‐up tests during piloting or even directly in production facilities. Recent examples of lifeline analysis (Haringa *et al*., 2016; Kuschel *et al*., 2017) should encourage similar applications in the future.

Predictions for large‐scale performance may be exploited in different ways: on the one hand, one may derive design criteria for the process or the bioreactor set‐up to prevent non‐wanted performance losses. On the other hand, detailed scale‐up studies may be used to engineer production strains such that non‐changeable process heterogeneities do no longer alter the performance of the cells. Recently, Michalowski *et al*. (2017) succeeded to engineer *E. coli* HGT which – among others – has the inherent property of constant intracellular *ppGpp* pools. *ppGpp*, the alarmone of the stringent response, is typically produced under glucose or nitrogen (ammonia) limitation. Accordingly, the stringent response is immediately induced when cells face shortage, as it may frequently occur in poorly mixed bioreactor zones. Strains with a constant *ppGpp* pool interrupt the intracellular signal transduction which makes them ‘blind’ for external changes. In addition, transcriptional dynamics as observed by Loffler *et al*. (2016) may be further exploited to identify frequently changing gene expressions with no apparent function for productivity. Those may serve as deletion candidates for saving expression energy thereby reducing the maintenance demand of the cells.

The rigorous application of these novel scale‐up concepts offers a series of economic benefits. Savings may occur (i) while transferring the process faster from the lab to the production, (ii) by achieving better performance in large scale and (iii) by reducing operation and investment costs for large‐scale fermenters (Noorman and Heijnen, 2017).

Regarding (i), considering that piloting or production tests often cost 40 000–80 000 €/run, one may easily calculate total savings assuming that the usual number of tests ranging from 10–15 reduces to 2–3 instead. Coincidentally, development times should be cut tremendously, because checks in piloting facilities often hamper the speed of bioprocess development. The proportional reduction of development times seems to be reasonable. Performance losses according to (ii) comprise not only less efficient substrate‐to‐product conversion but also increasing impurities in the fermentation broth and reduced amounts of product per batch. All factors deteriorate the economic success of the product. Their cumulative effect may easily sum up to 25%–50% of manufacturing costs on average. Accordingly, applying modern tools for scale‐up can secure the economic survival of the new process. Considering (iii), the use of robust production strains with distinct qualities for large‐scale application further allows to reduce OPEX (expenses for operation) and CAPEX (expenses for investment) likewise. Often, energy costs for mixing, mass transfer and cooling represent more than 10% of manufacturing costs in aerobic processes. Apparently, this fraction can be reduced if strains tolerate heterogeneities better than they do today. By analogy, investments in engines, compressors, pumps, gearing and stirrers may be reduced which will be reflected in product cost estimation by reduced depreciation. The latter typically contributes by another 10% of manufacturing costs for commodities.

## Conflict of interest

None declared.
